# Accurate monitoring of substrate-dependent growth reveals ecotypic differentiation among marine yeasts

**DOI:** 10.1093/ismeco/ycag010

**Published:** 2026-01-15

**Authors:** Berin Sena Arslan-Gatz, Mikkel Schultz-Johansen, Tom-Niklas Hollwedel, Sofie Niggemeier, Daniel Bartosik, Sreelakshmi Lakshmanan, Rolf Nimzyk, Antje Wichels, Gunnar Gerdts, Jan-Hendrik Hehemann, Tilmann Harder, Marlis Reich

**Affiliations:** MARUM, FB2, Molecular Ecology, University of Bremen, Leobener Str. 10, 28359 Bremen, Bremen, Germany; MARUM, FB2, Marine Glycobiology, University of Bremen, James Watt Str. 1, 28359 Bremen, Bremen, Germany; MARUM, FB2, Marine Chemistry, University of Bremen, James Watt Str. 1, 28359 Bremen, Bremen, Germany; MARUM, FB2, Marine Glycobiology, University of Bremen, James Watt Str. 1, 28359 Bremen, Bremen, Germany; Pharmaceutical Biotechnology, University of Greifswald, Friedrich-Ludwig-Jahn-Str. 17, 17489 Greifswald, Mecklenburg Western-Pomerania, Germany; MARUM, FB2, Molecular Ecology, University of Bremen, Leobener Str. 10, 28359 Bremen, Bremen, Germany; MARUM, FB2, Molecular Ecology, University of Bremen, Leobener Str. 10, 28359 Bremen, Bremen, Germany; FB2, Microbial Ecophysiology Group, University of Bremen, Leobener Str. 2, 28359 Bremen, Bremen, Germany; Biologische Anstalt Helgoland, Alfred Wegener Institute Helmholtz Center for Polar and Marine Research, Postfach 180, 27483 Helgoland, Schleswig-Holstein, Germany; Biologische Anstalt Helgoland, Alfred Wegener Institute Helmholtz Center for Polar and Marine Research, Postfach 180, 27483 Helgoland, Schleswig-Holstein, Germany; MARUM, FB2, Marine Glycobiology, University of Bremen, James Watt Str. 1, 28359 Bremen, Bremen, Germany; MARUM, FB2, Marine Chemistry, University of Bremen, James Watt Str. 1, 28359 Bremen, Bremen, Germany; Ecological Chemistry, Alfred Wegener Institute Helmholtz Center for Polar and Marine Research, Am Handelshafen 12, 27570 Bremerhaven, Bremen, Germany; MARUM, FB2, Molecular Ecology, University of Bremen, Leobener Str. 10, 28359 Bremen, Bremen, Germany

**Keywords:** mycoplankton, microbial loop, carbon turnover, DOM, laminarin, marine fungi, chemical diversity, degradation

## Abstract

Phytoplankton-derived dissolved organic matter (DOM) sustains complex marine microbial communities, yet the role of marine fungi—particularly yeasts—remains understudied regarding their substrate preferences, enzymatic strategies, and ecological relevance. We developed a novel protocol to investigate substrate-specific growth of marine fungal isolates under defined conditions and high temporal resolution. Using the β-1,3-glucan laminarin—a major marine storage polysaccharide of phytoplankton—and its oligomeric and monomeric breakdown products, we characterized growth and substrate utilization profiles of eleven marine yeast isolates from the epipelagic zone at Helgoland Roads, North Sea. Statistical analyses of growth kinetics distinguished four ecotypes with distinct substrate utilization patterns, quantified via phenol–sulfuric acid assays. Fluorophore-assisted carbohydrate electrophoresis (FACE) revealed the lack of endo-laminarinase activity, suggesting laminarin degradation depends on exo-acting enzymes. FACE also revealed a high diversity of short-chained laminarin-based intermediates accumulating over time, demonstrating that yeasts enhance chemical complexity during laminarin degradation and may fuel other microbes within the microbial loop. Representatives of each yeast ecotype were found to match abundant operational taxonomic units (OTU) in sequence similarity analyses of epipelagic mycoplankton datasets. This supports their ecological success and diverse substrate strategies. Rather than acting solely as opportunists, these yeasts may actively shape DOM turnover and carbon cycling within the microbial loop. Our study highlights a robust experimental approach for resolving functional diversity among marine yeasts and underpins their potential role in maintaining chemical diversity and substrate cross-feeding in the microbial loop.

## Graphical Abstract

**Graphical Abstract:**
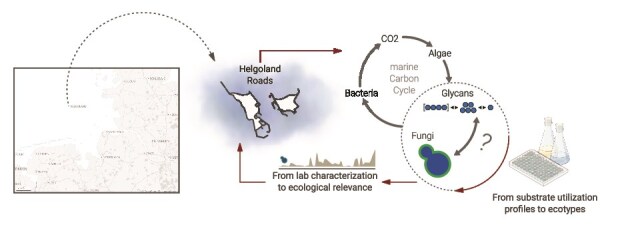

Copyright: OpenStreetMap contributors. Data licensed under the Open dAtabase Lecense (ODbL) v1.0.

## Introduction

Marine phytoplankton produce a diverse pool of organic material, including structurally simple and more complex glycans [1]. Simple glycans are immediately utilized and transformed at the base of the food web by heterotrophic microbial consortia [2], whereas complex glycans persist longer or even escape complete degradation [3]. This process, known as the “microbial loop” [4], involves assimilation and respiration of organic matter and is essential for transferring otherwise inaccessible carbon and energy to higher trophic levels.

Fresh primary-produced organic matter occurs in different stages of solution, adsorption, and aggregation and constitutes the main substrate for heterotrophic microplankton. During and post phytoplankton blooms the microbial community dynamics are largely structured by competitive or cooperative substrate utilization [5].

The bacterial contribution to the microbial loop is well studied, and reproducible succession patterns of specific bacterial clades have been observed and explained by substrate-induced forcing [6]. The bacterial decomposition strategies, species-specific enzyme repertoires [7], and feeding styles [8] strongly control the role of the individual bacterial members. In contrast, the role of marine saprotrophic fungi has long been neglected as potential players of the microbial loop, despite their ubiquitous distribution, functional diversity, and abundance [9–11]. Based on few studies, seasonal dynamics of marine fungi correlate positively with nutrients and organic matter, whereas the presence of interaction partners (e.g. phytoplankton, zooplankton, and bacteria) can have either a positive or negative impact [12–14]. During phytoplankton blooms, it has been shown that yeasts reach high biomass [15] and therefore likely play ecologically relevant roles in the microbial cycle. The identification of specific carbohydrate-active fungal enzymes (CAZymes) with high functional diversity in global pelagic fungal -omic datasets, as well as stable-isotope probing (SIP) analyses [16, 17], lend further support to the active involvement of fungi in the microbial loop.

To understand the influence and role of individual microorganisms in the marine carbon cycle, it is essential to correlate the chemical nature of organic substrates and their biochemical utilization [18]. Understanding the specific roles of individual microbial loop members requires an integrated approach that combines environmental analyses with targeted laboratory studies of single isolates, allowing results from artificial settings to be verified in complex natural environments.

Compared to bacteria, controlled *in vitro* cultivation studies of marine fungi under defined growth regimes and media compositions are lacking (but see [9, 19, 20]), especially for ecologically relevant fungal members in natural microbial communities. One goal of this study was to develop reproducible, time-resolved methods to detect substrate-specific fungal growth by adjusting culture conditions while keeping the medium consistent. The optimization workflow considered that fungi may grow on intracellular storage products [21] and tested various nutrient ratios [22] and the pre-activation of fungal metabolic pathways that may enhance the decomposition of specific glycans [23]. Another objective was to optimize the experimental growth protocol to correlate organic substrate size. High-molecular-weight (HMW) laminarin was used as ecologically relevant model organic substrate in accordance with [24]. This polysaccharide is a major constituent of phytoplankton blooms, accounting for a quarter of the annual marine primary production and three-digit micromolar concentrations in ambient seawater [25, 26]. We further included partially degraded laminarin subunits and monomeric glucose to temporally resolve the degradation of these substrates by marine yeast isolates.

## Materials and methods

### Fungal isolates

Eleven marine yeasts were isolated from surface water (1 m) at Helgoland Roads (North Sea, Germany, 54°11.3′ N, 7°54.0′ E) and stored in strain collections of M. Reich, A. Wichels, and G. Gerdts [27] (Supplementary Material Table S1). The ITS (internal transcribed spacer) regions were amplified using the primer pair ITS1f/ITS4 (5′-CTTGGTCATTTAGAGGAAGTAA-3′ [28]/5′-TCCTCCGCTTATTGATATGC-3′ [29] following the protocol of Yang *et al*. [30]. Amplicons were Sanger-sequenced at Eurofins Genomics (Ebersberg, Germany). Sequence quality was checked by manually inspecting pherograms, accepting only sequences with well-defined peak resolution. If needed, sequences were trimmed by a max. of 30 bp at the start and the end using the MEGA12 program [31]. For taxonomic classification, the quality-controlled ITS sequences were subjected to BLASTn analyses against the general FASTA release for eukaryotes v9.0 database of UNITE [32] incorporated as “SequenceID”-tool on the Global Biodiversity Information Facility (GBIF) website (https://www.gbif.org/tools/sequence-id; accessed 20250506). Taxonomic classification was performed with >80% query coverage and an e-value of 0 at the species level with >97% sequence identity or at the genus level with >90% sequence identity [33].

### Precultures of isolates

**Figure 1:**
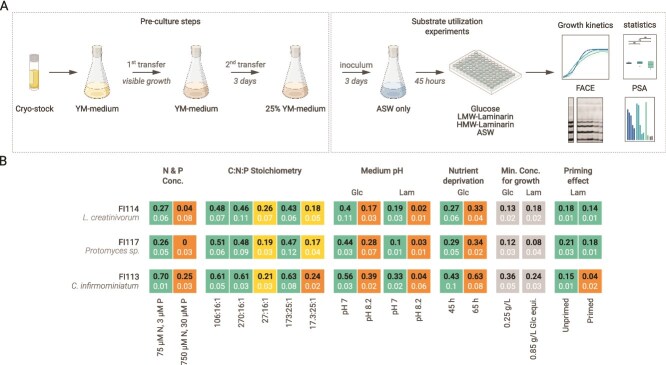
Experimental workflow. (A) Yeast isolates were cultivated from cryostocks using a defined multi-step pre-culture procedure before tracking and quantifying isolate-specific substrate turnover (Created in BioRender. Bartosik, D. (2025), Agreement numbers: PU28GRIUPC, SR28GRIUWF, DY28GRIV2G, QA28GRIV8A). (B) A modular approach was developed to evaluate substrate-specific growth in microtitre plates by examining the influence of various growth parameters on the ODmax values (mean + SD, *n* = 4) of three yeast isolates. Significant differences across conditions of a parameter are highlighted in differently coloured boxes (Kruskal–Wallis test, Dunn’s test, Bonferroni correction, *P* ≤ .05), while the same-coloured boxes indicate no significance between the two compared parameters (*P* > .05). Greyish-coloured boxes indicate ODmax values at the minimal glycan concentration at which growth was detectable. SD, standard deviation; Glc, glucose; Lam, HMW laminarin; equi., equivalents.

Precultures were prepared from glycerol cryostocks in YM medium [34] with artificial seawater (ASW) [35], containing 100 mg/ml each of ampicillin and kanamycin (Roth, Karlsruhe, Germany) and grown at 16°C under 13:11 h day:night at 150 rpm on a KL 2 shaker (Edmund Bühler GmbH, Tübingen, Germany) until visible growth. Precultures were inoculated at 1:1 ratio into fresh medium and, after 3 d, diluted into 25% YM medium total of 20 ml. After another 3 d, cultures were used as inoculum (Fig. 1A).

### Size definition of laminarin and carbohydrate quantification in laminarin-based media

A stock solution of 1.8 g/l laminarin (>96% purity, *Laminaria digitata,* L9634, Sigma-Aldrich®, Germany) was size-fractionated with spin column filters (Sartorius, Göttingen, Germany) of 5 and 10 kDa. The working solution used in all experiments contained laminarin in the size range between 5 and 10 kDa (defined as HMW laminarin). The carbohydrate concentration in HMW laminarin media was determined by phenol-sulfuric acid (PSA) [36]. Briefly, 550 μl of HMW laminarin were transferred to 1.5 ml tubes, topped with 367 μl 10 M hydrochloric acid, and carefully vortexed. Samples were hydrolysed at 100°C for 2 h under orbital shaking at 600 rpm (ThermoMixer C, Eppendorf, Hamburg, Germany). After cooling, 120 μl of the hydrolysate was topped with 600 μl of 95% sulfuric acid, heated to 90°C for 15 min at 900 rpm, followed by addition of 120 μl 5% aqueous phenol. The mixture was vortexed for 2 min, stored for 1 h at 4°C, after which absorbance of three replicates was measured at 490 nm in a plate reader (Clariostar Plus, BMG Labtech, Germany) and correlated with a calibration series of 0.02–0.5 g/l glucose. Due to the naturally occurring variability in the length and branching pattern of laminarin, calibration must be repeated for every new batch. For this project, two batches were used in total.

### Growth experiments

Growth experiments were conducted in 96-well plates (CELLSTAR, Greiner Bio-One GmbH, Frickenhausen, Germany) in 200 μl wells at 16°C and 260 rpm (CMS1000, Cerillo, Charlottesville, VA, USA) for 70 h and with replications (*n* = 4). The initial optical density (OD) was set to 0.1. Twelve wells served as ASW blanks. Growth on intracellular storage products was measured in ASW without further additions and monitored by automated OD recordings at 600 nm over 65 h at 30-min intervals using an ALTO plate reader (Cerillo) (Fig. 1A). Plates were inspected for biofilm formation and sampled at three time points (T) during the active growth phase to detect mat formation of yeast cells. T1 was taken at the start of the active growth phase, T2 midway through the active growth, and T3 shortly before the stationary phase [37].

The culture protocols were optimized with yeast isolates *Cystofilobasidium infirmominiatum* (FI113), *Lecuosporidium scottii* (FI114), and *Protomyces* (FI117). Preliminary growth experiments were carried out with 2.5 g/l glucose (Roth). The other conditions were adapted sequentially to build on each other and are listed below in the order of adjustment (Fig. 1B):

1. **N and P concentrations:** The required concentrations of inorganic nitrogen (N) and phosphorous (P) for yeast growth were tested. Specifically, concentrations 10 and 100 times higher than the yearly mean for N and P at Helgoland Roads [38] were tested: 75 and 3 μM, and 750 and 30 μM of NaNO_3_ and NaH_2_PO_4_, respectively.
2. **Elemental C:N:P ratio:** The fungal utilization of organic carbon may be influenced by the C:N:P ratio of growth media. Media were tested at Redfield (106:16:1, [39]), as well as high carbon (270:16:1) and low carbon (27:16:1) while maintaining the Redfield ratio for N:P. Additionally, media were adjusted to a 10× higher concentration than the N:P ratio in Helgoland waters (25:1) [40] with high carbon (173:25:1), and low carbon (17.3:25:1). The P concentration was 3 μM as determined in (condition 1). High and low carbon ratios were mimicked with glucose concentrations of 2.5 and 0.25 g/l, respectively.
3. **Growth medium pH:** As the environmental pH value affects extracellular enzymes, such as laminarinase [41], yeast growth was compared at pH 8.2 with enriched ASW (EASW, [42]) and pH 7.0 with ASW.
4. **Nutrient deprivation:** To distinguish yeast growth with and without addition of organic substrates, nutrient deprivation steps of 45 and 65 h were compared. Precultures (7.5 ml) at OD 0.6–1.2, representing max 200 μl, were transferred into ASW (7.5 ml) to achieve a final OD of 0.4.
5. **Glycan concentrations:** To determine detection thresholds in media containing glucose or HMW laminarin, growth was measured in a glucose concentration series from 0.00025 to 25 g/l. The detection threshold of HMW laminarin was tested at 0.017, 0.17, and 0.97 g/l, corresponding to 0.025, 0.25, and 0.85 g/l glucose equivalents, as determined by PSA.
6. **Priming effect:** Because enzymatic degradation of complex polysaccharides is energetically costly, we compared yeast growth under “primed” (pre-exposed to the substrate) and “nonprimed” (substrate added only at experiment start) conditions. For priming, 0.0017 g/l HMW laminarin was added during nutrient deprivation. Growth tests were then performed at the lowest HMW laminarin concentration where all isolates still showed detectable growth (see Condition 5).

### Preparation of low-molecular-weight laminarin

Two laminarinases, FaGH17A and FbGH30 [43], were used to partially digest HMW laminarin prior to growth experiments. FaGH17A is an endo enzyme targeting β-1,3-linkages, while FbGH30 cleaves the β-1,6-linked branch along the laminarin backbone. Both enzymes have high specificity at pH 7.0 [41]. A phosphate-buffered saline (137 mM NaCl, 2.7 mM KCl, 10 mM Na_2_HPO_4_, 1.8 mM KH_2_PO_4_) HMW laminarin stock solution (4 g/l) at pH 7.0 was hydrolysed for 16 h at room temperature (RT) with 0.5 μM of each enzyme in a final volume of 1.6 ml. Enzymes were inactivated at 98°C for 10 min, followed by centrifugation at 16 000 × g for 10 min at 4°C. The supernatant containing hydrolysed LMW laminarin, mainly composed of laminari-hexaose, -pentaose, -tetraose, and -biose (as evidenced by FACE, see Tracking and Quantification of Substrates and Intermediate Degradation Products), was sterile-filtered, quantified by PSA, and diluted to a working concentration of 0.85 g/l glucose equivalents.

### Yeast growth experiments with high-molecular-weight and low-molecular-weight laminarin as single carbon source

The growth of 11 yeast isolates on HMW and LMW laminarin as the only carbon source at a normalized concentration of 0.85 g/l glucose equivalents (PSA) was compared to a negative ASW control. The positive control contained 0.85 g/l glucose equivalent to 0.97 g/l HMW laminarin in the size range between 5 and 10 kDa, corresponding to 30–60 glucose units linked by mainly β-1,3 and some β-1,6 glycosidic bonds.

### Tracking and quantification of substrates and intermediate degradation products

Based on their different growth kinetics, four functional ecotypes were distinguished among all isolates. One isolate of each ecotype was used (selection on isolate with highest ODmax on glucose) to follow substrate degradation and identify intermediate products, namely, *Metschnikowia bicuspidata* (FI018, Group 1), *Cy. infirmominiatum* (FI113, Group 2), *Candida sake* (FI270, Group 3), and *Tausonia pullulans* (FI475, Group 4). Samples were quantified by PSA and profiled by fluorophore-assisted carbohydrate electrophoresis (FACE) (Fig. 1A). In addition to HMW and LMW laminarin, two laminarin oligosaccharides (laminarihexaose and laminaribiose; Megazyme, Auchincruive, UK) were tracked using the standard growth protocol. Specifically, subsamples of 25 μl were taken at time 0 (T0), 9 (T1), 22 (T2), 45 (T3), and 70 h (T4). The enzyme activity was immediately quenched at 99°C for 10 min, followed by drying under vacuum, redissolution in 5 μl ultrapure water, and derivatization for FACE [44]. Briefly, 1 μl 0.02 M 8-aminoaphtalene-1,3,6-trisulfonic acid (ANTS) and 2 μl 1 M NaBH_3_CN were added to each sample and the reaction mixture was incubated overnight at 37°C. Three microliters of 100% glycerol were added, and 8 μl of the final mix was loaded onto a standard acrylamide gel (35%). Electrophoresis was performed at 100 V for 30 min, followed by 200 V at 4°C using precooled running buffer (25 mM Tris-base, 250 mM glycine). An oligosaccharide standard was prepared by combining 0.85 mg/ml laminarihexaose, 0.85 mg/ml laminaripentaose, 0.56 mg/ml laminaritetraose, and 0.43 mg/ml laminaribiose.

### Matching of yeast isolates with operational taxonomic units (OTUs) of environmental datasets

For each yeast isolate, the V7/V8 regions of the 18S ribosomal DNA (rDNA) gene sequence were amplified using the primer pair FF390/FR-1 (5′-CGATAACGAACGAGACCT-3′/5′-ANCCATTCAATCGGTANT-3′ [45]), following Banos *et al*. [46]. Sequencing and quality-control followed the one for ITS (Fungal Isolates section). Isolates’ sequences were then compared to the 18S rDNA gene datasets of Banos *et al*. [12] and Priest *et al*. [15], which used the same primer set. The two datasets monitored the mycoplankton community of Helgoland Roads over the course of 2015/2016 and during the spring phytoplankton bloom in 2017, respectively (for details, see Supplementary Material S1). These datasets functioned as a subject database against which the isolate sequences were queried via the BLASTn function of the BLAST [47] command line application (https://www.ncbi.nlm.nih.gov/books/NBK279690/; accessed 20250625). Only BLASTn hits with an e-value <1e-150, a query coverage >95%, and a percent identity >98% were considered to belong to the same species as the queried isolate [48, 49].

### Statistics

The computer program AMIGA (download 2024-12-05) [50] was used to analyze growth data. Growth curves were modelled by nonparametric Gaussian Process (GP) regression approach to minimize technical variability and fluctuations across four different replicates. Next, the modelled growth curve on intracellular storage products (negative control) and the carbon source provided was checked for significant differences using a Bayesian test with default settings [51]. In the case of significant differences, the growth values on the substrates were normalized by subtracting the growth values of the negative control. Finally, the maximal OD (ODmax) was read out from the normalized substrate-specific growth models.

To test differences due to growth conditions or substrate-specific growth, a Kruskal–Wallis test [52] was performed on individual growth parameters and substrates, followed by Dunn’s test of multiple comparisons using a *P*-value with Bonferroni correction of ≤.05 as a threshold for statistical significance. Both tests were run with the RStudio program [53] v 2024.04.0 + 764 using the core functions as well as the “Dunn.test” v.1.3.6. (https://CRAN.R-project.org/package=dunn.test, accessed 20250526). Graphs were designed using the package “ggplot2” [54].

## Results

### Taxonomy of fungal isolates

BLASTn analyses resolved eight isolates at the species level and linked them to a species hypothesis (SH) in the UNITE database [55]. However, only seven of the eight SHs contained sufficient information for taxonomic classification at the species level. The remaining three isolates were resolved at the genus level. Five isolates were Ascomycota, namely, *Meyerozyma guiliermondii* (FI145), *Ca. sake* (FI270), *M. bicuspidata* (FI018), *Pezizomycotina* sp. (FI122), and *Protomyces* (FI117). Six isolates belonged to the Basidiomycota, namely, *Cy. infirmominiatum* (FI113), *Kondoa* (FI425), *Leucosporidium scottii* (FI114), *Rhodotorula mucilaginosa* (FI127), *T. pullulans* (FI475), and *Vishniacozyma victoriae* (FI121) (Table 1).

**Table 1:** Taxonomic information of the 11 yeast isolates and their classification into ecotypes based on their substrate utilization strategies of HMW and LMW laminarin and glucose (statistics on ODmax values, see also Fig. 2). A, Ascomycota; B, Basidiomycota; functional ecotypes: ODmax values between substrates showed: 1, no significance; 2, no significance between HMW and LMW laminarin; 3, no significance between LMW laminarin and glucose; 4, significance between all three substrates (Dunn’s test, Bonferroni-corrected *P* < .05). *Present in surface water of Helgoland Roads in 2011 [27]; occurrence patterns, see also Fig. 3. +forming own subgroup due to lack of growth on laminarin. NA, not applicable.

Isolate ID	Taxonomy	Species hypothesis	Taxonomy	Ecotype	Matched with	Category of Banos *et al*./Priest *et al*. occurrence patterns [12]
FI018	*Metschnikowia bicuspidata*	NA	Saccharomycetes (A)	1	Y/N	IV—long-lasting
FI113	*Cystofilobasidium infirmominiatum*	SH1295646.09FU	Tremellomycetes (B)	2	N/N*	NA
FI121	*Vishniacozyma victoriae*	SH0994206.09FU	Tremellomycetes (B)	3	Y/Y	IV—long-lasting
FI127	*Rhodotorula mucilaginosa*	SH1008722.09FU	Microbotryomycetes (B)	3	Y/Y	IV—long-lasting
FI270	*Candida sake*	SH1040972.09FU	Saccharomycetes (A)	3	Y/Y	I—boom-bust like
FI425	*Kondoa*	Agaricostilbomycetes (B)	3	Y/Y	III—steady	
FI114	*Leucosporidium creatinivorum*	SH4274207.09FU	Microbotryomycetes (B)	4	Y/Y	IV—long-lasting
FI117	*Protomyces*	Taphrinomycetes (A)	4	Y/Y	III—steady	
FI122	*Pezizomycotina* sp.	SH0960948.09FU	(A)	4	Y/N	II—frequent-peaking
FI475	*Tausonia pullulans*	SH0753078.10FU	Tremellomycetes (B)	4	Y/Y	II—frequent-peaking
FI145	*Meyerozyma guiliermondii*	SH1029393.09FU	Saccharomycetes (A)	4+	Y/Y	II—frequent-peaking

### Growth parameters

A significant difference in growth parameters was observed (Dunn’s test) for the three isolates *Cy. infirmominiatum* (FI113), *L. scottii* (FI114) and *Protomyces* (FI117) showing Bonferroni-corrected *P*-values of <.05. For details, see Fig. 1B and Table S2.

1. **N and P concentrations:** Preliminary growth assays showed significantly higher ODmax at lower nutrient concentrations (75 and 3 μM NaNO3/ NaH2PO4; ODmax 0.26–0.69) compared to higher levels (750 and 30 μM; ODmax 0–0.24).
2. **Elemental C:N:P ratio:** Significant variation of growth based on different C:N:P ratios was only observed between high- and low-glucose stoichiometries across isolates. ODmax values were 0.43–0.63 for high-glucose stoichiometries (106, 270, and 173) and 0.17–0.26 for low-glucose stoichiometries (27 and 17.3). However, an exception was *Cy. infirmominiatium* (FI113), which exhibited significant differences between the two low-glucose stoichiometries with ODmax of 0.21 and 0.24, respectively.
3. **Growth medium pH:** All isolates had significantly higher ODmax at pH 7.0 (glucose: 0.39–0.56; laminarin: 0.10–0.33) than at pH 8.2 (glucose: 0.17–0.39; laminarin: 0.02–0.04).
4. **Nutrient deprivation** for 65 h significantly increased ODmax (0.33–0.63) compared to 45 h (0.27–0.43).
5. **Glycan concentrations:** Growth on glucose was significantly higher than controls but decreased at 25 g/l (0.21–0.62) compared to 2.5 g/l (0.32–0.77). *L. scottii* and *Protomyces* did not grow below 0.25 g/l (0.12–0.13), while *Cy. infirmominiatum* grew even at 0.0025 g/l (0.06). At low HMW laminarin concentration (0.017 g/l), no significant growth (vs. control) was observed; only at 0.97 g/l HMW laminarin did significant growth occur (0.26–0.80).
6. **Priming effect:** All isolates had higher ODmax in unprimed (0.15–0.21) vs. primed conditions (0.04–0.18), but significance was only seen for *Cy. infirmominiatum* (*P* < .01).

### Optimal growth protocol

The preliminary growth tests including three isolates suggested running cultivations in 96-well plates over 70 h with ASW as base medium, supplemented with 75 μM NaNO_3_ and 3 μM NaH_2_PO_4_, and no further adjustment of the C:N:P ratio. Fungal precultures were nutrient-deprived for 45 h without any further priming prior to growth experiments on different carbon sources. To ensure optimal growth of all yeast isolates, 0.85 g/l glucose equivalents of HMW and LMW laminarin were used, while the corresponding glucose equivalent served as positive control. Growth in 96-well plates did not lead to biofilm or mat-formation by yeast cells.

### Yeast growth on glucose, high-molecular-weight and low-molecular-weight laminarin, and oligosaccharides

All isolates grew significantly better on glucose (Bayesian test) than on intracellular storage products, with ODmax values ranging from 0.32 to 0.61. Ten isolates grew significantly on HMW laminarin with ODmax values ranging from 0.1 to 0.37, with the exception of *T. pullulans* (FI145, ODmax = 0). All isolates grew significantly better on LMW laminarin with ODmax values of 0.14–0.48 than on intracellular storage products (Supplementary Material Table S3).

**Figure 2:**
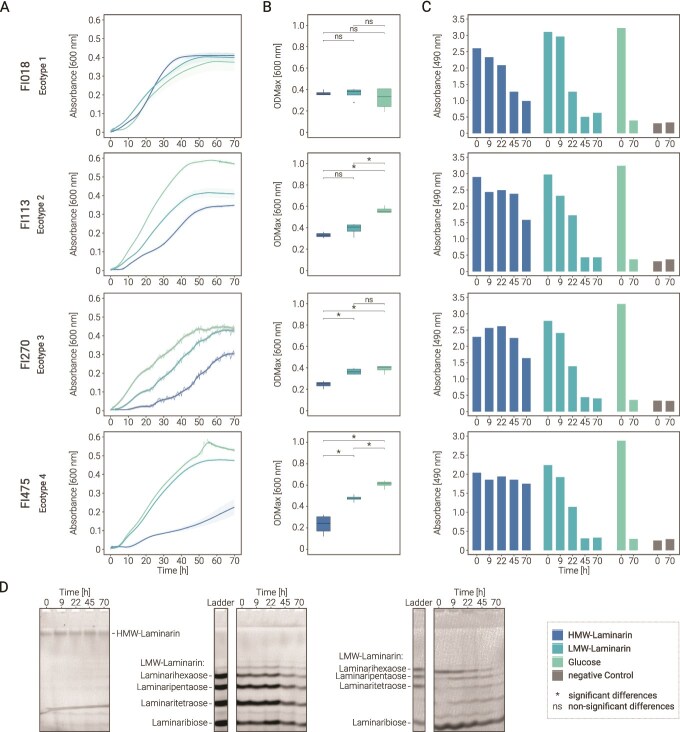
Ecotype-specific time-dependent substrate turnover. Panels show data for each ecotype: (A) growth kinetics, (B) significance in substrate-specific growth (Kruskal–Wallis test (*P* ≤ .05), Dunn’s test with Bonferroni-corrected *P* ≤ .05), and (C) substrate tracking in medium via PSA. (D) FACE degradation patterns of different substrates, shown for one representative isolate (FI475). HMW laminarin (D.1); laminari-hexaose and -biose (D.2); LMW laminarin (D.2).

Based on their substrate-specific growth patterns, the yeast isolates were grouped into four ecotypes: (1) no significant growth differences among substrates; (2) significantly better growth on glucose than on HMW and LMW laminarin; (3) no significant difference between growth on glucose and LMW laminarin; and (4/4*) significant differences across all substrates (Bonferroni-corrected *P* < .05) with *M. guiliermondii* (FI145) forming its own subgroup 4* with no growth on laminarin (Table 1, Fig. 2A and B, Supplementary Material Table S3).

No differences in decomposition and intermediate products were observed between representative isolates of each group (Fig. 2). As quantitatively revealed by PSA (Fig. 2C) and qualitatively by FACE (Fig. 2D), the intensity of the HMW laminarin band decreased slightly across time points T0–T4 (Fig. 2D.1). However, no intermediate degradation products were visible at any time point, suggesting that HMW laminarin was digested by yeast-derived exo-acting glycosylhydrolases. In contrast, the LMW laminarin mixture, mainly consisting of laminari-hexaose, -pentaose, -tetraose, -triose to -biose, evenly decreased over the observation time frame (Fig. 2D.2). Degradation of the defined oligosaccharide mixture, consisting of laminari-hexaose and -biose, revealed increasingly smaller intermediates over time, especially laminari-hexaose was stepwise degraded via -pentaose into -tetraose and -triose (Fig. 2D.3).

### Correlation of isolates in environmental datasets

**Figure 3:**
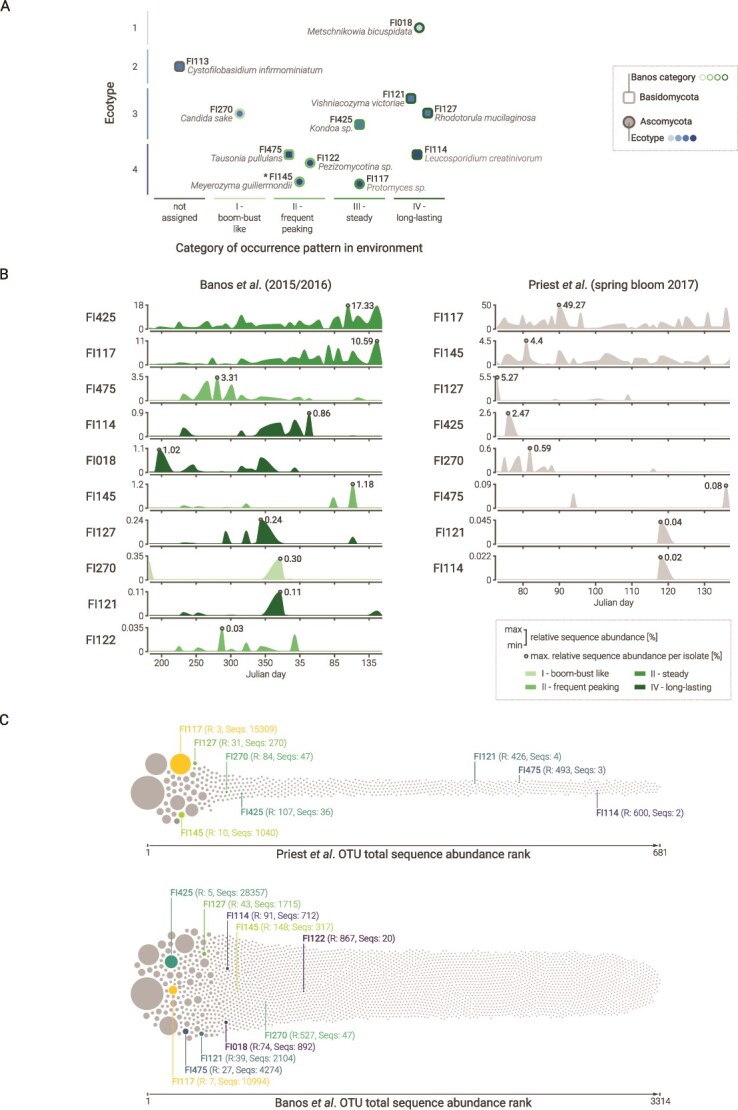
Occurrence of isolates in surface water of Helgoland Roads (North Sea, Germany). (A) Relationship between functional ecotype, taxonomy, and categories of occurrence patterns over the 2015/2016 annual cycle ([12]). (B) Occurrence patterns based on relative sequence abundance of isolate matching OTUs throughout the course of the year in 2015/2016 (Banos *et al*. data) and during the spring 2017 phytoplankton bloom (Priest *et al*. data). (C) The abundance rank [Rx, based on total sequence abundance (Seqs:x)] of the isolate matching OTUs relative to the total fungal OTUs in the two mycoplankton communities. Each isolate is represented by a specific colour.

All isolates, except *Cy. infirmominiatum* (FI113), were detected in the Banos *et al*. [12] dataset (Fig. 3A and B). In that study, OTUs and thus the matching isolates were grouped into four types based on temporal abundance: (I) boom-bust, with one to three short peaks (plateaus ≤2 weeks); (II) frequent peaking, with multiple peaks (>3) or a single long plateau (≥3 weeks); (III) steady, showing continuous presence with moderate fluctuation; and (IV) long-lasting, with at least two peaks and plateaus of 2 weeks or more (Fig. 3A and B).

Seven isolates were detected in the Priest *et al*. dataset of [15] (Fig. 3C), except *Cy. infirmominiatum* (FI113), *M. bicuspidata* (FI018), and *Pezizomycotina* sp. (FI122).

Ecotype 1 showed similar growth on all substrates and belonged to the 0.2% most abundant OTUs (defined on absolute sequence abundance) in the Banos *et al*. dataset. It was classified as a long-lasting type (IV). Its highest relative abundance was 1% in a sample from 16 July 2015. Ecotype 2 was not detected in the Banos *et al*. or Priest *et al*. datasets, but it had been observed at Helgoland Roads in April 2011 and 2013.

Ecotype 3 grew poorly on laminarin but performed better on glucose and LMW laminarin. These isolates were among the top 0.7% most abundant OTUs in Banos *et al*., showing variable dynamics. Their maximum relative sequence abundances (0.3%–17.3%) occurred throughout 2015/2016. In the Priest *et al*. dataset, they ranked within the top 5% of OTUs. Their highest relative abundances (0.01%–5.4%) were mainly recorded during the early spring phytoplankton bloom in March. Ecotype 4 grew best on glucose and showed clear substrate-dependent differences. In Banos *et al*., isolates ranked among the top 0.8% most abundant OTUs, with many showing frequent peaks (Type II). Their highest relative abundances (0.03%–10.6%) did not follow a specific seasonal pattern. In the Priest *et al*. dataset, they ranked among the top 4.1% of OTUs, with the highest abundances (0.01%–44.7%) occurring during the later phase of the spring phytoplankton bloom (Fig. 3B and C).

## Discussion

### Differentiation of substrate-specific growth among marine fungal isolates

Microtitre plate growth experiments with marine fungi hold promise to uncover substrate utilization patterns and, when paired with integrated chemical and omics analyses, offer a powerful means to clarify the role of marine fungi in the marine carbon cycle. Because such experiments are rare (see [20, 56]), the rationale for this study was to develop a robust and scalable growth assay to capture species-specific substrate utilization patterns at high temporal resolution, distinguishing fungal growth on external substrates from the mobilization of intracellular storage products [21]. Isolates were selected for the development of the growth assay to reflect taxonomic diversity: one Ascomycota and two Basidiomycota (Microbotryomycetes and Tremellomycetes). The optimization phase demonstrated a high degree of consistency across almost all individual growth conditions, so no further isolates were tested.

So far, little research has examined how the C:N:P stoichiometry of media affects marine fungal growth. In terrestrial fungi, taxonomy and guild composition have been shown to influence stoichiometry [57]. Since Redfield stoichiometry applies to many plankton species [58], we investigated its relevance to marine mycoplankton. Using NaNO_3_ and NaH_2_PO_4_ as N and P sources, we found that the Redfield ratio did not significantly increase ODmax values. Instead, statistical differences were related to the substrate’s absolute carbon content. This aligns with previous findings on aquatic stream fungi, where the lability of the carbon source influences both growth rate and biomass stoichiometry [59, 60]. Elevated dissolved inorganic N in the medium has also been shown to enhance growth rates without altering biomass C:N ratios [60]. In our study, N and P concentrations were elevated 10-fold above environmental levels to ensure nutrient sufficiency, particularly given the high carbon concentrations required for OD measurements. Notably, increased ODmax was observed only at higher carbon concentrations, not uniformly across treatments with the highest N ratios. However, the extent to which elevated nutrient concentrations influenced these results versus reflecting inherent stoichiometric traits of fungal biomass, as reported for diverse species and environments [57], remains unresolved.

The priming effect may mask substrate-specific fungal growth. Of the three tested isolates, growth without prior priming achieved higher ODmax values with laminarin, although this was only significant for one isolate. The priming effect, particularly for glycan degradation, has been shown to rely on multiple factors, including phylogeny, substrate type, or the availability of other nutrients [61]. These complex interactions may explain why unprimed conditions may yield more consistent or enhanced growth responses [62]. One outcome and recommendation when adopting fungal growth protocols is to test for potential priming effects before testing new isolates and/or growth substrates. The optimized workflow enabled simultaneous analyses of growth substrates and growth kinetics, ensuring efficient timing and parameter readout. Its modular adaptation may facilitate rapid adjustments to other fungal species and growth-determining factors.

### Potential role and ecotypic niche differentiation of fungi in the marine microbial loop

Pelagic bacterial communities are distinguished by enzymatic repertoires and feeding strategies and differ in turnover abilities of net primary-produced dissolved and particulate organic matter [63]. The corresponding bacterial ecotypes thus vary in their abilities to utilize different organic matter, influencing their efficiency and interaction within the microbial loop. In analogy, the yeast isolates examined in this study also revealed distinct catabolic responses to the substrates used. Based on their different substrate utilization patterns and growth kinetics, the yeast isolates were categorized into four groups, suggested to represent distinct ecotypes. These ecotypes likely represent ecological niche adaptations. Indeed, representatives of different ecotypes were among the most abundant OTUs in the environmental amplicon dataset of Helgoland Roads, suggesting that several fungal ecotypes are relevant in this highly competitive environment.

While sequence matching between environmental OTUs and isolates relied on 18S rDNA similarity (≥98% threshold), which is robust for many yeast species [48, 49], false-positive matches cannot be entirely ruled out. However, the spatial and temporal alignment of environmental and isolate data, despite FI018 and FI127 originating from nearby Helgoland waters, supports the reliability of our OTU–isolate matches.

The ubiquitous presence of laminarin in many parts of the global surface ocean, its rapid turnover and the widespread expression of laminarinase activity across the ocean suggest its crucial role in the marine carbon cycle [25]. Laminarin degradation by bacteria is rapid, surpassing rates observed for other polysaccharides [8, 64]. In particular, bacterial laminarinase activity is thought to drive its degradation [6, 65]. SIP analyses [66] and global meta-transcriptomic studies [16] demonstrated that marine fungi process phytoplankton-derived polymers like laminarin, with widespread fungal glycoside hydrolase (GH) activity for β-1,3-linked glucan degradation in the world’s oceans.

Most fungi possess β-(1,3)-glucanases, enzymes capable of degrading the β-(1,3)-glucan laminarin due to the presence of fungal β-(1,3)-glucans in their own cell walls [67], yet marine isolates exhibit lower enzymatic activity compared to terrestrial fungi [68, 69]. While high laminarin turnover is typically linked to specialized lifestyles like phyto-parasites [70], which are in the oceans, mainly zoosporic fungi [9], the significant increase in saprotrophic yeast biomass during the phytoplankton bloom in spring 2017 in Helgoland Roads, a period characterized by high laminarin availability [15], is all the more surprising. This apparent contradiction suggests that marine yeasts may process laminarin via alternative or yet unidentified strategies. However, it cannot be ruled out that they use other glycans available during the phytoplankton bloom [24].

To occupy a niche within the microbial loop, yeasts must either cooperatively or competitively degrade and take up substrates. The isolate *M. bicuspidata* (FI018), capable of using all tested substrates (Ecotype 1) and effectively degrading HMW laminarin, was among the 0.2% most abundant OTUs persisting across different seasons in the surface waters of Helgoland Roads, likely due to its substrate versatility. Bacteria with competitive laminarin uptake possess diverse strategies, such as direct binding of laminarin to the outer cell wall [71], as well as unregulated expressions of involved polysaccharide utilization units (PULs) enabling quick responses to bloom conditions [24]. The uptake strategies by *M. biscuspidata* (FI018) remain unclear, as no endoenzymes for polymer degradation was detected by FACE. A possible control measure could be effective transport systems [72] coupled with/or the proximity of CAZymes and membrane transporters on the fungal cell wall [73].

In contrast, members of Ecotype 4 showed significantly better growth on monomeric glucose. Over half of their corresponding OTUs exhibited rapid, seasonal bursts at Helgoland Roads in 2015/16, accounting for 0.8% of the most abundant OTUs. Many of these OTUs were also highly abundant during the spring phytoplankton bloom in 2017. Spring phytoplankton blooms significantly increases polymeric substrate hydrolysis, rapidly producing LMW products [8] and boosting the abundance of LMW substrate consumers [74, 75]. Some gammaproteobacterial clades specialize in metabolizing small organic substrates, including sugar oligomers and monomers [72], thus filling a niche in this highly competitive environment. Our findings suggest that certain yeast groups may effectively occupy this niche, paralleling the metabolic strategies of bacterial counterparts. Furthermore, yeasts not only consume LMW products but also enhance their diversity through degradation, contributing to the chemical diversity within the microbial loop. Future studies should quantify the contributions of bacteria and yeast in this ecological niche.

## Conclusion

The modular approach developed in this study provides a scalable tool for advancing marine fungal ecology research. By integrating chemical, physiological, and microbial analyses, it supports interdisciplinary workflows and accelerates discovery. Combined with amplicon or metagenome studies, this approach links community-level patterns with mechanistic insights from single-isolate experiments, enhancing our understanding of fungal contributions to glycan turnover and carbon sequestration in the ocean.

Our findings highlight marine yeasts as previously underestimated contributors to the microbial loop, revealing unexpectedly high ecotype diversity among epipelagic yeasts. Each ecotype exhibits distinct strategies for utilizing laminarin and its breakdown products, ranging from metabolic generalists to specialists with narrower substrate preferences. These strategies enhance chemical diversity through degradation activities, with fungal competitiveness deriving from either substrate flexibility or efficient uptake systems for oligosaccharides and monosaccharides.

While fungal β-glucans are well characterized structurally, the genetic and enzymatic mechanisms enabling marine fungi to degrade these polysaccharides remain poorly understood. Future integration of transcriptomic, genomic, and biochemical approaches will be essential to unlocking the metabolic potential of marine fungi and defining their roles in oceanic carbon dynamics, offering new insights into microbial ecology and biogeochemical cycling in a changing ocean.

## Supplementary Material

Supplementary_Material_ycag010

## Data Availability

All data generated in this project are provided in the form of tables and graphs in the main manuscript and the Supplementary Material. Sequence data of the isolates can be accessed via the International Nucleotide Sequence Database Collaboration (INSDC) databases, e.g. the European Nucleotide Archive (ENA, https://www.ebi.ac.uk/ena/browser/home) with the project accession number PRJEB100540. As the isolates are currently only deposited in the laboratory’s own culture collection, anyone interested should contact Marlis Reich directly.
