# Supplementary material for: Accurate monitoring of substrate-dependent growth reveals ecotypic differentiation among marine yeasts
Source: ISME Commun. 2026 Jan 15;6(1):ycag010. doi: 10.1093/ismeco/ycag010 (PMC12903948; doi:10.1093/ismeco/ycag010)
Supplement: Supplementary_Material_ycag010 [file supplementary_material_ycag010.docx]

**Supplementary Material**

# Text S1: Description of the sampling strategy used for Banos *et al.* and Priest *et al.*

Seawater samples for the datasets of Banos *et al*. (2020, DOI: 10.3389/fmicb.2020.01305) and Priest *et al*. (2021, DOI: 10.1111/1462-2920.15331) were collected at the same station as for this study, which is the Long-term Ecological Research (LTER) station “Kabeltonne” at Helgoland Roads (Wiltshire et al., 2010, DOI:10.1007/s12237-009-9228-y), located about 70 km off the mainland in the German Bight of the North Sea (Germany, 54° 11.3’ N, 7° 54.0’ E). Samples were collected on board of the research vessel Aade, with an impeller pump (Jabsco, USA) from ~1 m-depth into a sterile 10 L bottle (Nalgene, Germany) and were directly processed in the laboratory of the Biologische Anstalt Helgoland.

For the Banos *et al.* dataset, water samples were taken once a week (from July 2015 to June 2016, resulting in a total of 43 samples. Two liters of the water was filtered directly without pre-filtration onto a Sterivex GP filter unit (0.22 µm PES membrane, Merck, Darmstadt, Germany) using a peristaltic pump (Verder, Germany).

For the Priest *et al*. dataset, 41 samples were taken in total during the period between March and May 2017, spanning all stages of the spring phytoplankton bloom, some by daily sampling. At each time point, 1 L of seawater was sequentially filtered through 10 µm, 3 µm and 0.2 µm isopore polycarbonate filters using a vacuum pump (Merck Millipore, Darmstadt, Germany). DNA was extracted from 0.2 and 3 µm polycarbonate filters from each time point.

**Table S1: Information on isolation time point, medium and corresponding reference of the eleven yeast isolates.** All isolates were members of the microbial communities of the surface waters (1 m) of Helgoland.

**Isolate ID Isolation source Sampling date Isolation date Isolation medium culture collection**

**Surface waters (1 m)**

FI018 water above *Fucus vesiculosus* 20210520 20210530 1% F. vesiculosus extract Reich lab

patch, (54°10.45N, 7°53.23E) solution in ASW^1^

FI113 Helgoland Roads (54°11.30N, 7°54.00E) 20110414 20110414 Wickerham’s YM^2^ _4_

FI121 Helgoland Roads (54°11.30N, 7°54.00E) 20110414 20110414 Wickerham’s YM^2^ _4_

FI127 Helgoland Roads (54°11.30N, 7°54.00E), 20110414 20110429 Wickerham’s YM^2^ _4_

after 2 weeks incubation at pH7.67

FI270 Helgoland Roads (54°11.30N, 7°54.00E) 20220425 20220726 Wickerham’s YM^2^ Reich lab

FI425 Helgoland Roads (54°11.30N, 7°54.00E) 20220425 20220817 Wickerham’s YM^2^ Reich lab

FI114 Helgoland Roads (54°11.30N, 7°54.00E) 20110414 20110414 Wickerham’s YM^2^ _4_

FI117 Helgoland Roads (54°11.30N, 7°54.00E) 20110414 20110414 Wickerham’s YM^2^ _4_

FI122 Helgoland Roads (54°11.30N, 7°54.00E) 20110414 20110414 Wickerham’s YM^2^ _4_

FI145 Helgoland Roads (54°11.30N, 7°54.00E) 20211005 20220715 T5^3^ Reich lab

FI475 Helgoland Roads (54°11.30N, 7°54.00E) 20110414 20110414 Wickerham’s YM^2^ _4_

^1^ASW, ^35,41^

^2^Wickerham’s YM: 0.3 % yeast extract, 0.3 % malt extract, 0.5 % peptone, 1.0 % dextrose, and 2.0 % agar in sterile surface seawater of Helgoland Roads, pH 6.2, supplemented with 250 mg/l chloramphenicol (Sigma)

^3^T5: Sigma sea salts 40g/l, NAG (Sigma A3286) 2 g/l, Guillard’s F/2 (Sigma G9903) 20 ml/L, agar for microbiology (Sigma) 15 g/l, prepared in milliQ-H_2_O and amended with 100 mg/l chloramphenicol.

^4^Krause *et al.*, 2013, DOI: 10.3354/Ame01622

**Table S2:** **Optimal growth parameters.** Only growth with a significant difference to growth on internal storage products (Bayesian test) were analyzed by Kruskal-Wallis (K-W) followed by Dunn’s test. The test results are represented with P values, “ns” indicates P > 0.05.

**Condition 1: N and P concentrations**

**Isolates Sµbstrate comparison K-W test Dµnn’s test**

FI113 750 µM N_30 µM P vs. 75 µM N_3 µM P *P = 0.02 P = 0.01*

FI114 750 µM N_30 µM P vs. 75 µM N_3 µM P *P = 0.02 P = 0.01*

FI117 750 µM N_30 µM P vs. 75 µM N_3 µM P *P = 0.02 P = 0.01*

**Condition 2: Elemental C:N:P ratio**

**Isolates Substrate comparison K-W test Dunn’s test**

FI113 condition 2 P = 0.01

FI113 106:16:1 vs. 270:16:1 ns

FI113 106:16:1 vs. 27:16:1 *P =* 0.01

FI113 106:16:1 vs. 173:25:1 ns

FI113 106:16:1 vs. 17.3:25:1 *p* = 0.01

FI113 270:16:1 vs. 27:16:1 *P =* 0.01

FI113 270:16:1 vs. 173:25:1 ns

FI113 270:16:1 vs. 17.3:25:1 *P =* 0.01

FI113 27:16:1 vs. 173:25:1 *P =* 0.01

FI113 27:16:1 vs. 17.3:25:1 *P =* 0.04,

FI113 173:25:1 vs 17.3:25:1 *P =*  0.01

FI114 condition 2 P = 0.01

FI114 106:16:1 vs. 270:16:1 ns

FI114 106:16:1 vs. 27:16:1 *P =* 0.01

FI114 106:16:1 vs. 173:25:1 ns

FI114 106:16:1 vs. 17.3:25:1 *P =* 0.01

FI114 270:16:1 vs. 27:16:1 *P =* 0.01

FI114 270:16:1 vs. 173:25:1 ns

FI114 270:16:1 vs. 17.3:25:1 *P =* 0.01

FI114 27:16:1 vs. 173:25:1 *P =* 0.01

FI114 27:16:1 vs. 17.3:25:1 ns

FI114 173:25:1 vs17.3:25:1 *P =*  0.01

FI117 condition 2 P =0.01

FI117 106:16:1 vs. 270:16:1 ns

FI117 106:16:1 vs. 27:16:1 *P =* 0.01

FI117 106:16:1 vs. 173:25:1 ns

FI117 106:16:1 vs. 17.3:25:1 *P =*  0.01

FI117 270:16:1 vs. 27:16:1 *P =* 0.01

FI117 270:16:1 vs. 173:25:1 ns

FI117 270:16:1 vs. 17.3:25:1 *P =*  0.01

FI117 27:16:1 vs. 173:25:1 *P =*  0.01

FI117 27:16:1 vs. 17.3:25:1 ns

FI117 173:25:1 vs 17.3:25:1 *P =*  0.01

**Condition 3: Growth medium pH**

**Isolates Substrate comparison K-W test Dunn’s test**

FI113 Glucose pH 7 vs. pH 8.2 *P = 0.3* *P = 0.01*

FI114 Glucose pH 7 vs. pH 8.2 *P = 0.04* *P = 0.01*

FI117 Glucose pH 7 vs. pH 8.2 *P = 0.03* *P = 0.01*

FI113 Laminarin pH 7 vs. pH 8.2 *P = 0.03* *P = 0.01*

FI114 Laminarin pH 7 vs. pH 8.2 *P = 0.04* *P = 0.01*

FI117 Laminarin pH 7 vs. pH 8.2 *P = 0.03* *P = 0.01*

**Condition 4: Nutrient deprivation**

**Isolates Substrate comparison K-W test Dunn’s test**

FI113 No-deprivation vs. 45h-deprivation *P = 0.01 P = 0.02*

FI114 No-deprivation vs. 45h-deprivation *P = 0.02* *P = 0.01*

FI117 No-deprivation vs. 45h-deprivation *P = 0.01* *P = 0.00*

FI113 No-deprivation vs. 65h-deprivation *P = 0.01 P = 0.01*

FI114 No-deprivation vs. 65h-deprivation *P = 0.02* *P = 0.01*

FI117 No-deprivation vs. 65h-deprivation *P = 0.01* *P = 0.01*

**Condition 5: Glycan concentrations** were tested by analysing at which carbon concentrations growth was detectable. As a carbon source served glucose or HMW laminarin. Significant values in the Bayesian test are shown with an asterisk (*), indicating that the growth on this substrate was significantly different from the growth of internal storage products.

**Isolates Substrate ODmax**

FI013 Glucose_25g/L 0.56*

FI114 Glucose_25g/L 0.21*

FI117 Glucose_25g/L 0.22*

FI113 Glucose_2.5g/L 0.78*

FI114 Glucose_2.5g/L 0.33*

FI117 Glucose_2.5g/L 0.34*

FI113 Glucose_0.25g/L 0.25*

FI114 Glucose_0.25g/L 0.13*

FI117 Glucose_0.25g/L 0.18*

FI113 Glucose_0.025g/L 0.05*

FI114 Glucose_0.025g/L 0

FI117 Glucose_0.025g/L 0

FI113 Glucose_0.0025g/L 0.04*

FI114 Glucose_0.0025g/L 0.01

FI117 Glucose_0.0025g/L 0

FI113 Glucose_0.00025g/L 0.23*

FI114 Glucose_0.00025g/L 0.02

FI117 Glucose_0.00025g/L 0

FI113 HMW laminarin_0.17g/L 0

FI114 HMW laminarin_0.17g/ 0.01

FI117 HMW laminarin_0.17g/ 0.03*

FI113 HMW laminarin _0.97g/L 0.26*

FI114 HMW laminarin _0.97g/L 0.18*

FI117 HMW laminarin _0.97g/L 0.08*

**Condition F: Priming effect**

**Isolates Substrate comparison K-W test Dunn’s test**

FI113 Unprimed vs. Primed_laminarin P *= 0.02 P = 0.01*

FI114 Unprimed vs. Primed_laminarin ns ns

FI117 Unprimed vs. Primed_laminarin ns ns

**Table S3: Substrate utilization profiles of yeast isolates.** Isolate- and substrate-specific ODmax values (mean ± S.D., n = 4), all of which were significantly different from growth on internal energy reserves (Bayesian test) with one exception. Using statistical analysis (Kruskal-Wallis test (P < 0.05) followed by Dunn's test with Bonferroni correction (P < 0.05)) based on ODmax differences, isolates were classified into four functional ecotypes*. Significant differences are indicated by different letters. #, no significant difference to growth on isolate’s internal energy reserves. *In ecotype 4, FI145 formed own subgroup due to lack of growth on laminarin.

**Isolate Functional ODmax**

**ecotype Glucose LMW laminarin HMW laminarin**

FI018 1 0.31 ± 0.08 0.37 ± 0.02 0.37 ± 0.0

FI113 2 0.56^a^ ± 0.01 0.39^b^ ± 0.04 0.33^b^ ± 0.0

FI121 3 0.36^a^ ± 0.05 0.40^a^ ± 0.03 0.29^b^ ± 0.01

FI127 3 0.35 ^a^ ± 0.03 0.30^a^ ± 0.0 0.16^b^ ± 0.0

FI270 3 0.39 ^a^ ± 0.01 0.37^a^ ± 0.0 0.25^b^ ± 0.0

FI114 4 0.39^a^ ± 0.09 0.25^b^ ± 0.01 0.19^c^ ± 0.01

FI117 4 0.44^a^ ± 0.02 0.20^b^ ± 0.03 0.10^c^ ± 0.01

FI122 4 0.56^a^ ± 0.05 0.30^b^ ± 0.03 0.12^c^ ± 0.0

FI475 4 0.61^a^ ± 0.03 0.48^b^ ± 0.02 0.23^c^ ± 0.08

FI145 4* 0.43^a^ ± 0.12 0.14^b^ ± 0.0 0^c#^ ± 0.00
